# The Value of Herbarium Collections to the Discovery of Novel Treatments for Alzheimer’s Disease, a Case Made With the Genus *Eriodictyon*

**DOI:** 10.3389/fphar.2020.00208

**Published:** 2020-03-10

**Authors:** Pamela Maher, Wolfgang Fischer, Zhibin Liang, David Soriano-Castell, Antonio F. M. Pinto, Jon Rebman, Antonio Currais

**Affiliations:** ^1^Cellular Neurobiology Laboratory, Salk Institute for Biological Studies, La Jolla, CA, United States; ^2^Mass Spectrometry Core, Salk Institute for Biological Studies, La Jolla, CA, United States; ^3^Department of Botany, San Diego Natural History Museum, San Diego, CA, United States

**Keywords:** Alzheimer’s disease, herbarium, neurodegenerative disorders, neuroprotection, traditional medicine California

## Abstract

Plants, in particular those with a history in traditional medicine, hold enormous potential as sources of new therapies for dementias such as Alzheimer’s disease (AD). The largest collections of plants can be found in herbaria all over the world, but the value of these collections to AD drug discovery has been significantly neglected. As a proof of principle, we investigated the neuroprotective activity of herbarium specimens of *Eriodictyon* (yerba santa), a genus with a long history of usage by the indigenous tribes in California to treat respiratory and age-related complications. Dichloromethane extracts were prepared from leaves of 14 *Eriodictyon* taxa preserved in the SD Herbarium located at the San Diego Natural History Museum. The extracts were tested for neuroprotection in nerve cells against oxytosis and ferroptosis and for anti-inflammatory activity in brain microglial cells exposed to bacterial lipopolysaccharide. In parallel, the levels of the flavanones sterubin, eriodictyol and homoeriodictyol were measured by mass spectrometry. Several *Eriodictyon* species presented strong neuroprotective and anti-inflammatory activities. The protective properties of the extracts correlated with the amount of sterubin, but not with eriodictyol or homoeriodictyol, indicating that sterubin is the major active compound in these species. The occurrence of eriodictyol and homoeriodictyol may be predictive of the phylogenetic relationship between members in the genus *Eriodictyon*. The data offer insight into the traditional use of yerba santa across indigenous tribes in California, while demonstrating the value of herbarium collections for the discovery of novel therapeutic compounds for the treatment of neurodegenerative diseases.

## Introduction

Alzheimer’s disease (AD) is the most common form of neurodegenerative dementia among the elderly. It affects millions of people worldwide and it has serious deleterious consequences for the lives of patients and caregivers alike. There are no drugs for AD that are disease modifying in the sense that they slow down or reverse the progression of the neurodegeneration process. This is due in part to a lack of therapies developed based upon the primary drivers of the disease. Because old age is the greatest risk factor for AD, we have been arguing that it is critical to consider the detrimental processes that take place with aging as therapeutic targets for AD (Prior et al., 2014; Currais, 2015; Schubert et al., 2018). Based on this premise, our laboratories have developed a number of cell culture models to study nerve cell death pathways that mimic different toxicities and stresses associated with the aging brain and AD (Prior et al., 2014). In the past 10 years, we have used these assays to successfully identify new AD drug candidates that are extremely protective in animal models of dementia by preventing specific aspects of the aging process in the brain (Currais et al., 2014b, 2015, 2017, 2019; Goldberg et al., 2018).

Recognizing the potential of our approach for drug discovery and considering the urgent need for new compounds with improved therapeutic activities, we have embraced the endeavor of searching for additional sources of such compounds. Because our current AD drug candidates are either derivatives of natural compounds or natural compounds themselves (Prior et al., 2014; Schubert et al., 2018), our search has been focused on nature’s pharmacopeia. In fact, traditional herbal medicines have long been used all over the world to treat disorders related to the nervous system. In 2014, we showed for the first time that our screening approach could be used to directly study extracts of plants with ethnopharmacological significance and identify the main active components (Currais et al., 2014a). These findings led us recently to screen a large library of extracts from plants with known pharmacological properties, representing 367 unique plant species and 304 unique genera native to diverse parts of the world (Fischer et al., 2019). Analysis of one of the most potent extracts identified the flavanone sterubin from the plant *Eriodictyon californicum* (Hook. & Arn.) Torr. (Boraginaceae family) as a highly neuroprotective and anti-inflammatory compound. We are now testing sterubin in animal models to determine its drug-like characteristics and toxicity.

Interestingly, *E. californicum*, commonly known as yerba santa (“sacred herb”), is a plant native to California that was used by indigenous American tribes to treat an array of illnesses, some directly associated with aging, including rheumatism, headaches, inflammation and respiratory complications such as asthma, cough, and lung infections (Moerman, 2009). The discovery of a plant with medicinal value in our “backyard” prompted us to question whether other such plants exist that could be sources of previously unidentified neuroprotective compounds. Indeed, the ethnobotanical history of the American West is rich, with numerous species frequently included in traditional health practices (Anderson, 2005; Moerman, 2009). However, to screen all these plants in our assays would require a voluminous effort of identification and collection. Therefore, we have partnered with the Botany Department at the San Diego Natural History Museum (SDNHM). Given that the Department holds a herbarium collection of nearly 275,000 specimens of native and naturalized plants mostly of the southwestern United States and northwestern Mexico, we sought to find out whether small amounts of material from the preserved specimens (some well over 100 years old) could be used to identify compounds with translational potential to treat AD.

Given our previous findings regarding the neuroprotective properties of *E. californicum* (Fischer et al., 2019), we reasoned that this species would serve as a good case study. In addition, given that other species of the genus *Eriodictyon* display a distinct geographical distribution, yet they were also used in traditional medicine by indigenous tribes, we extended our screening to all species of the genus. The levels of sterubin as well as two of the other main flavonoids (eriodictyol and homoeriodictyol) present in *E. californicum* (Bacon et al., 1986; Fischer et al., 2019) were measured. We hypothesized that if sterubin is a major active component in *E. californicum* responsible for the protection in our assays (Fischer et al., 2019), its presence and therapeutic properties might help explain the medicinal value attributed to the other members of the genus *Eriodictyon*.

## Materials and Methods

All reagents were obtained from Sigma-Aldrich (St. Louis, MO, United States), unless otherwise stated. Eriodictyol and homoeriodictyol were purchased from Indofine Chemical Company (Hillsborough, NJ, United States). Sterubin was a gift from Jakob Ley at Symrise AG (Holzminden, Germany).

### Plant Material

Plant material used in this study was removed from voucher specimens that are deposited and accessioned in the SD Herbarium located at SDNHM. Herbarium specimens are preserved plant pieces (typically stems with leaves and reproductive parts) or entire plants that are pressed and dried and have associated data (e.g., locality, date of collection, collector, etc.) used for scientific study. The specimens are stored in standard, sealed herbarium cabinets that have a 45–55% relative humidity within the cabinets. The cabinets are located in a pest-controlled room that is kept at 18–21°C. All plant specimens in the SD Herbarium have been legally collected from natural areas. Identification and verification of herbarium specimens in the SD Herbarium are conducted mostly by the SDNHM Curator of Botany. The nomenclature used in this study for the *Eriodictyon* taxa follows The Jepson eFlora and The Plant List (http://ucjeps.berkeley.edu/eflora, http://www.theplantlist.org, accessed on December 8, 2019) (Table 1). Permission for sampling of the herbarium specimens was granted by the SDNHM Curator of Botany.

**TABLE 1:** Descriptive information relative to the *Eriodictyon* taxa studied.

Accession number	Species and variety	Common name	Collection date	Country	State	District
38515	*E. californicum* (Hook. & Arn.) Decne.	California Yerba Santa	5/25/1933	United States	California	San Mateo
249224	*E. altissimum* P.V. Wells	Indian Knob Mountainbalm	5/23/1978	United States	California	San Luis Obispo
139563	*E. angustifolium* Nutt.	Narrow-leaved Yerba Santa	6/28/1996	Mexico	Baja California	Ensenada
248184	*E. trichocalyx* A. Heller var. *trichocalyx*	Hairy Yerba Santa	5/18/2014	United States	California	San Bernardino
126445	*E. capitatum* Eastw.	Lompoc Yerba Santa	6/6/1967	United States	California	Santa Barbara
216133	*E. crassifolium* Benth. var. *crassifolium*	Thick-leaved Yerba Santa	4/13/2009	United States	California	San Diego
222888	*E. sessilifolium* Greene	Sessile-leaved Yerba Santa	3/20/2012	Mexico	Baja California	Ensenada
146246	*E. trichocalyx* A. Heller var. *trichocalyx*	Hairy Yerba Santa	5/25/2000	United States	California	San Bernardino
6696	*E. trichocalyx* A. Heller var. *trichocalyx*	Hairy Yerba Santa	1878	United States	California	San Diego
269686	*E. tomentosum* Benth.	Woolly Yerba Santa	7/14/2009	United States	California	Fresno
262493	*E. trichocalyx* A. Heller var. *trichocalyx*	Hairy Yerba Santa	5/2/2013	United States	California	San Diego
175829	*E. traskiae* subsp. *smithii* Munz	Smith’s Yerba Santa	6/10/2006	United States	California	Santa Barbara
38519	*E. crassifolium* var. *nigrescens* Brand	Bicolored Yerba Santa	6/23/1935	United States	California	Ventura
244830	*E. sessilifolium* Greene	Sessile-leaved Yerba Santa	7/13/2015	United States	California	San Diego
64071	*E. californicum* (Hook. & Arn.) Decne.	California Yerba Santa	7/25/1965	United States	California	Fresno
252348	*E. traskiae* Eastw. subsp. *traskiae*	Trask’s Yerba Santa	5/19/2000	United States	California	Los Angeles
186939	*E. trichocalyx* var. *lanatum* (Brand) Jeps.	San Diego Yerba Santa	4/21/2008	United States	California	San Diego
243158	*E. lobbii* (A. Gray) Greene	Matted Yerba Santa	8/14/2010	United States	California	Placer
206781	*E. parryi* (A. Gray) Greene	Poodle-dog Bush	7/8/2010	United States	California	San Diego

*Species are ordered according to Table 2, in order to facilitate comparison between both tables.*

### Preparation of Extracts

Dried mature plant leaves (1–3 leaves, the equivalent of 100–800 mg depending on the species) were carefully removed from herbarium specimens that had abundant material. The extracts were prepared according to the protocol used to generate the library of plant extracts tested in our previous study (Fischer et al., 2019). Briefly, leaves were crushed, and non-polar extracts prepared by steeping material in dichloromethane (DCM) (1 ml of DCM per 100 mg of material) overnight at room temperature with agitation. The following day, most of the DCM was removed using a rotary evaporator, and the residual DCM was completely evaporated in a fume cupboard overnight. The dried product was solubilized in dimethyl sulfoxide (DMSO) at 50 mg/ml. Insoluble materials were removed by centrifugation and discarded. Samples were frozen at −80°C for long-term storage. The extraction yields obtained, expressed as a percentage of dry weight, are shown in Table 2.

**TABLE 2:** Extraction yields, biological activities in the oxytosis, ferroptosis and inflammation assays, and metabolite levels (sterubin, eriodictyol, and homoeriodictyol) of extracts from the *Eriodictyon* species studied.

Accession number	Extraction yield (%)^a^	Oxytosis (1/EC_50_)^b^	Ferroptosis (1/EC_50_)^b^	Inflam. (1/EC_50_)^b^	Sterubin		Eriodictyol		Homoeriodictyol	
							
					pmol/μg^c^	%^d^	pmol/μg^c^	%^d^	pmol/μg^c^	%^d^
38515	5.81	2.381	0.444	0.417	597.38	1.048	2.52	0.004	398.66	0.667
249224	14.2	1.333	0.488	0.278	346.81	1.487	0.23	0.001	250.32	1.024
139563	12.78	0.800	0.625	0.370	402.99	1.555	1.65	0.006	203.82	0.750
248184	12.45	0.625	0.200	0.115	129.48	0.487	0.60	0.002	194.90	0.699
126445	4.55	0.333	0.111	0.081	34.29	0.047	n.d.^f^	n.d.^f^	n.d.^f^	n.d.^f^
216133	7.46	0.290	0.235	0.217	20.78	0.047	n.d.^f^	n.d.^f^	15.50	0.033
222888	6.25	0.100	0.200	0.085	n.d.^f^	n.d.^f^	n.d.^f^	n.d.^f^	n.d.^f^	n.d.^f^
146246	11.77	0.100	0.080	0.114	21.97	0.078	0.20	0.001	86.96	0.295
6696	3.57	0.083	0.049	0.080	24.00	0.026	0.08	<0.001	80	0.082
269686	8.77	0.080	0.071	0.067	n.d.^f^	n.d.^f^	n.d.^f^	n.d.^f^	n.d.^f^	n.d.^f^
262493	7.05	0.040	0.042	0.050	n.d.^f^	n.d.^f^	1.87	0.004	273.05	0.554
175829	8.68	0.040	0.040	0.145	n.d.^f^	n.d.^f^	n.d.^f^	n.d.^f^	n.d.^f^	n.d.^f^
38519	6.31	0.031	0.041	0.045	n.d.^f^	n.d.^f^	0.03	<0.001	125.03	0.227
244830	10.52	0.027	0.040	0.031	n.d.^f^	n.d.^f^	n.d.^f^	n.d.^f^	n.d.^f^	n.d.^f^
64071	8.22	0.027	0.027	0.063	n.d.^f^	n.d.^f^	2.83	0.007	373.01	0.883
252348	6.59	0.025	0.025	0.048	n.d.^f^	n.d.^f^	n.d.^f^	n.d.^f^	n.d.^f^	n.d.^f^
186939	5.39	0.020	0.022	0.065	n.d.^f^	n.d.^f^	0.92	0.002	247.93	0.385
243158	9.09	n.p.^e^	n.p.^e^	0.020	n.d.^f^	n.d.^f^	n.d.^f^	n.d.^f^	n.d.^f^	n.d.^f^
206781	10.4	2.000	0.526	0.278	n.d.^f^	n.d.^f^	n.d.^f^	n.d.^f^	n.d.^f^	n.d.^f^

*Taxa are ordered according to the level of protection of their respective extracts in the oxytosis assay (highest to lowest). *E. lobbii* (243158) and *E. parryi* (206781) are shown at the right end of the charts due to their phylogenetic distance to the other members of the *Eriodictyon* genus. ^*a*^Extraction yield (%), percentage of dried extract per dried leaf material; ^*b*^1/EC_50_, inverted half maximal effective concentration (EC_50_ units in μg/ml); ^*c*^pmol/μg, picomol of metabolite per microgram of dried DCM extract; ^*d*^%, percentage of metabolite in dried leaf [calculated based on the MW of metabolite, extraction yield and concentration of metabolite in DCM extract (pmol/μg)]; ^*e*^n.p., no protection; ^*f*^n.d., not detected.*

### Old-Age Associated Toxicity Assays

#### Cell Culture

HT22 mouse hippocampal nerve cells were cultured in high-glucose Dulbecco’s modified Eagle’s medium (DMEM) (Invitrogen, Carlsbad, CA, United States) supplemented with 10% fetal calf serum (FCS) (Hyclone, Logan, UT, United States), and incubated at 37°C in an atmosphere with 10% CO_2_. Mouse BV2 microglial cells were grown in low glucose DMEM supplemented with 10% FCS, and incubated in similar conditions.

#### Oxytosis

5 × 10^3^ HT22 cells were plated in 96-well plates. After 24 h of culture, the medium was exchanged with fresh medium and 5 mM glutamate and the indicated concentrations/dilutions of extracts were added. After 24 h of treatment, viability was measured by the 3-(4, 5-dimethylthiazolyl-2)-2,5-diphenyltetrazolium bromide (MTT) assay as previously described (Tan et al., 2001). In the absence of a neuroprotective compound ≥90% of the cells die under these conditions. In all cases, cells in the dishes were examined microscopically before the addition of the MTT reagent to ensure that any positive results in the MTT assay are not an artifact due to interaction of the extracts with the assay chemistry.

#### Ferroptosis

Cells were treated with 100 nM RSL3 alone or treated with RSL3 + extract and then assayed after 24 h for cell survival using the MTT assay. This concentration of RSL3 produces 90–95% cell death.

#### Inflammation

Mouse BV2 microglial cells were plated at 5 × 10^5^ cells in 35 mm tissue culture dishes. After growth overnight, the cells were treated with 25 μg/ml bacterial lipopolysaccharide (LPS) alone or in the presence of the extracts. After 24 h, the medium was removed, spun briefly to remove floating cells and 100 μl assayed for nitrite using 100 μl of the Griess Reagent in a 96 well plate. After incubation for 10 min at room temperature the absorbance at 550 nm was read on a microplate reader, as described previously (Fischer et al., 2019).

### Mass Spectrometry

Mass spectrometry quantification was performed in three independent runs and values were found to be similar. Plant extracts reconstituted in DMSO at a concentration of 50 mg/ml were diluted 1:10,000 in 0.1% aqueous formic acid. Analysis was performed by LCMS on a Dionex Ultimate 3000 LC system coupled to a TSQ Quantiva mass spectrometer (ThermoFischer). Five microliters of the diluted samples were injected and a gradient of 0.1% formic acid with increasing concentrations of acetonitrile (18–45% in 15 min) was run at a flow rate of 0.2 ml/min. The eluate was electrosprayed into the mass spectrometer. Multiple reaction monitoring (MRM) was performed for the three compounds sterubin, homoeriodictyol, and eriodictyol (sterubin: [M+H]^+^ 303.2, transitions: 167.1, 163.1, 145.0, 135.0; homoeriodictyol: [M+H]^+^ 303.2, transitions: 153.1, 177.1, 145.1, 179.1; eriodictyol: [M+H]^+^ 289.2, transitions: 153.0, 163.0, 145.0, 135.0). External standards for all three compounds were run at concentrations of 500 pM, 5 nM, 50 nM, and 500 nM.

### Statistical Analysis

The EC_50_’s were determined from sigmoidal dose response curves using GraphPad Prism 6. Experiments were done at least three independent times.

## Results

### Plant Selection and Extract Preparation

**FIGURE 1:**
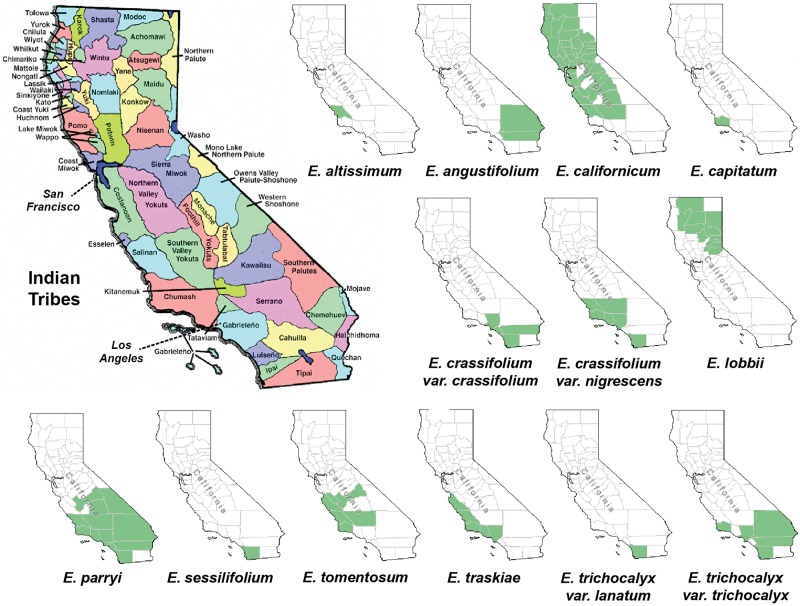
Maps of California depicting the Indian tribal territories (large map on the left) and the geographic distribution of each of the *Eriodictyon* species and infraspecies studied (green in smaller maps). Sources: California Indian Library Collections and United States Department of Agriculture, Natural Resources Conservation Service (USDA, NRCS) (https://plants.sc.egov.usda.gov/).

In order to find out whether plant specimens from the SD Herbarium collection could be used directly to identify novel neuroprotective compounds, we prepared and tested extracts from specimens of *E. californicum* as well as other *Eriodictyon* species. We have recently shown that an *E. californicum* extract is very neuroprotective and anti-inflammatory (Fischer et al., 2019), and the first aim here was to test if extracts prepared from small amounts of herbarium material would also be active after long-term preservation. In addition, because other members of the genus *Eriodictyon* have distinct geographical distributions (Figure 1) but were historically used in traditional medicine by indigenous tribes (Moerman, 2009), we also tested specimens from all other *Eriodictyon* species. In total, 19 specimens of 10 species including multiple varieties of two of these species (14 taxa) were studied: one *Eriodictyon altissimum* P.V. Wells, one *Eriodictyon angustifolium* Nutt., two *Eriodictyon californicum* (Hook. & Arn.) Decne., one *Eriodictyon capitatum* Eastw., one *Eriodictyon crassifolium* Benth. var. *crassifolium*, one *Eriodictyon crassifolium* var. *nigrescens* Brand, one *Eriodictyon lobbii* (A. Gray) Greene (recently changed from the genus *Nama*), one *Eriodictyon parryi* (A. Gray) Greene (previously changed from the genus *Turricula*), two *Eriodictyon sessilifolium* Greene, one *Eriodictyon tomentosum* Benth., one *Eriodictyon traskiae* subsp. *smithii* Munz, one *Eriodictyon traskiae* Eastw. subsp. *traskiae*, one *Eriodictyon trichocalyx* var. *lanatum* (Brand) Jeps., and four *Eriodictyon trichocalyx* A. Heller var. *trichocalyx* (Table 1).

The collection dates of the specimens studied ranged from 1878 to 2015 (Table 1). The plants were collected from multiple counties/municipal districts of the states of California (United States) and Baja California (Mexico) (Table 1), and deposited in the SD Herbarium. To minimize the impact on the preserved herbarium material, only 1–3 leaves (the equivalent of 100–800 mg dry weight) of each plant were removed from specimens where vegetative material was abundant. An extraction protocol based on that used to generate the library of plant extracts tested in our previous study (Fischer et al., 2019) was followed. The extraction yields ranged from 3.57 to 14.2%, varying with the years since collection (Table 2).

### Screen in Biological Assays

We then tested the biological activity of the extracts in our toxicity assays. The main screening model in our laboratory relies on the lethal induction of endogenous reactive oxygen species (ROS) in the HT22 hippocampal nerve cell line, a form of non-apoptotic programmed cell death called oxytosis with physiological features similar to those implicated in the nerve cell damage seen in AD (Tan et al., 2001; Chen et al., 2011; Chiruta et al., 2012; Currais and Maher, 2013; Prior et al., 2014). Oxytosis can be triggered by inhibiting cystine uptake via system Xc- with glutamate (Tan et al., 2001), leading to depletion of intracellular GSH, production of ROS and cell death. Brain GSH decreases with age in humans, and loss of GSH is associated with impaired cognitive function, lipid peroxidation, microglial activation, and endothelial dysfunction (Tan et al., 2001; Currais and Maher, 2013). Oxytosis resembles in many aspects the recently described mechanism of cell death called ferroptosis (Lewerenz et al., 2018). Ferroptosis can be specifically induced by inhibiting glutathione peroxidase 4 (GPX4) (Dixon et al., 2012; Yang et al., 2014) and has also been implicated in a number of pathological processes including neurodegenerative diseases (Lewerenz et al., 2018). Therefore, we tested the protection of our extracts in these two toxicity models.

**FIGURE 2:**
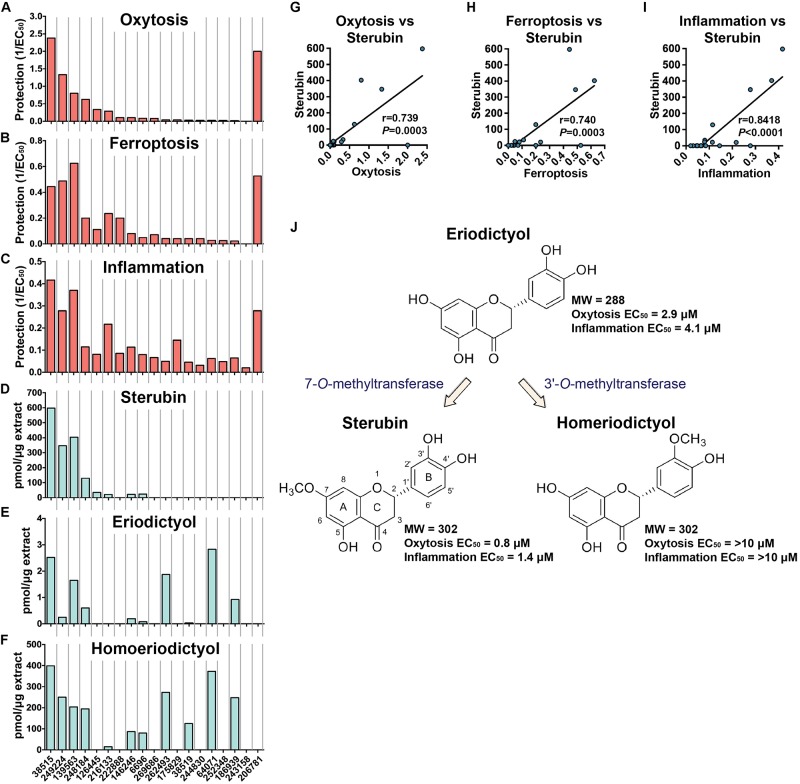
Biological protection and chemical information. Protection against oxytosis **(A)**, ferroptosis **(B)**, and inflammation **(C)** by the *Eriodictyon* extracts are indicated as inverted EC_50_ (1/EC_50_). Levels of sterubin **(D)**, eriodictyol **(E)**, and homoeriodictyol **(F)** are calculated as picomol per microgram of dried DCM extract (pmol/μg). Species are ordered from left to right according to Table 2. Correlation graphs between sterubin levels and protection against oxytosis **(G)**, ferroptosis **(H)**, and inflammation **(I)**. **(J)** Chemical structures and proposed biosynthetic pathway of sterubin, eriodictyol, and homoeriodictyol are shown, with respective MW and EC_50_ protection against oxytosis and inflammation.

The protection levels of each extract are indicated in Table 2 and Figures 2A,B. With the exception of *E. lobbii*, all species offered some level of protection against both toxicities, which seemed to correlate (Figures 2A,B). This correlation is likely explained by the mechanistic overlap between both toxicities (Lewerenz et al., 2018). Protection does not appear to be associated with any particular species; since extracts from *E. californicum* could be found as being the most protective (38515) and one of the least protective (64071). The same applies to *E. trichocalyx* and varieties, which showed protection levels spread across the chart (248184, 146246, 6696, 262493, and 186939).

Considering the traditional use of yerba santa to treat inflammatory conditions (Moerman, 2009), that sterubin itself is a potent anti-inflammatory (Fischer et al., 2019), and that inflammation is a major feature of AD (Wyss-Coray and Rogers, 2012), we also assessed the effects of the *Eriodictyon* extracts in a model of LPS-induced inflammation (Figure 2C). We found that the extracts presented strong anti-inflammatory activity that was similar in extent to their protection against oxytosis and ferroptosis.

Importantly, the length of time that the specimens were preserved in the herbarium did not appear to affect the quality of the material and its biological activities, as the two most protective extracts were prepared from plants that are among the oldest tested. This was the case for *E. californicum* that was collected in 1933 (38515) and *E. altissimum* from 1978 (249224).

### Flavonoid Measurement

In our recent publication, we showed that most of the protection afforded by *E. californicum* extracts in the assays was due to the production of sterubin, an extremely neuroprotective flavanone (Fischer et al., 2019). Since the *Eriodictyon*’s tested showed varied levels of protection against oxytosis and ferroptosis (Table 2 and Figures 2A,B), we asked whether this could be explained by distinct amounts of sterubin in the specimens. To address this question, we measured the levels of sterubin in the extracts by MS. We also measured two additional flavanones, eriodictyol and homoeriodictyol, which are characteristic of the genus *Eriodictyon* but that we have shown to be much less protective than sterubin (Fischer et al., 2019).

Sterubin was detected in eight of the extracts from six species – *E. californicum* (38515), *E. altissimum* (249224), *E. angustifolium* (139563), *E. trichocalyx* (248184, 146246, and 6696), *E. capitatum* (126445), and *E. crassifolium* (216133) (Table 2 and Figure 2D). The other extracts from some of these species did not present detectable levels of sterubin, indicating that its production varies within the same species. This was the case for *E. californicum* (38515 vs. 64071), *E. trichocalyx* (248184/146246/6696 vs. 262493/186939), and *E. crassifolium* (216133 vs. 38519). The gross amount of sterubin in the dried plant leaves reached considerable levels as high as 1.5% by weight in *E. altissimum* (249224) and *E. angustifolium* (139563) (Table 2 and Figure 2D).

Unlike sterubin, the occurrence of eriodictyol and homoeriodictyol was very consistent across species (Table 2 and Figures 2E,F). No eriodictyol and homoeriodictyol were found in *E. capitatum* (126445), *E. sessilifolium* (222888 and 244830), *E. tomentosum* (269686), *E. traskiae* (175829 and 252348), *E. lobbii* (243158), and *E. parryi* (206781). With the exception of *E. capitatum* (126445), no sterubin was present in these specimens either. The gross amount of eriodictyol in the dried leaves was extremely low (less than 0.007%) while the gross amount of homoeriodictyol reached 1% in *E. altissimum* (249224).

Strikingly, with the exception of *E. parryi* (206781), the levels of sterubin but not eriodictyol and homoeriodictyol, correlated with the protection against oxytosis and ferroptosis (Figures 2G,H, *r* = 0.739, *P* = 0.0003 and *r* = 0.740, *P* = 0.0003, respectively). That the levels of eriodictyol and homoeriodictyol do not correlate with neuroprotection in the assays is not surprising, as we have previously reported that the EC_50_ of sterubin (0.8 μM) is considerably lower than the EC_50_ of eriodictyol (2.9 μM) and homoeriodictyol (>10 μM) in this assay (Fischer et al., 2019). In addition, the levels of eriodictyol in the extracts tested were at least one order of magnitude lower than those of sterubin (Table 2). Moreover, we confirmed that, similar to oxytosis and ferroptosis, the anti-inflammatory activity in the extracts correlated with the amount of sterubin (Figure 2I, *r* = 0.8418, *P* < 0.0001). Together these data indicate that sterubin is a major active component across *Eriodictyon* species. The protection by *E. parryi* (206781), previously classified in the *Turricula* genus, may be explained by an as yet unidentified secondary metabolite(s).

Interestingly, the presence of homoeriodictyol appears to correlate with the presence of eriodictyol (Figures 2E,F). Given that the presence of a methoxy group on the 3′-position of the B ring in homoeriodictyol is the only distinction between the chemical structures of both flavanones (Figure 2J), it is likely that the conversion of eriodictyol into homoeriodictyol is mediated by a regiospecific *O*-methyltransferase (OMT) (3′-*O*-methyltransferase, 3′OMT). A methoxy group at the 7-position of the A ring is also the only difference between the chemical structure of sterubin and eriodictyol, raising the possibility that the conversion of eriodictyol into sterubin is mediated by another regiospecific OMT (7-*O*-methyltransferase, 7OMT). The fact that sterubin was only detected in a subset of the extracts that have eriodictyol and homoeriodictyol, confirms the possible involvement of a distinct 7-*O*-methyltransferase (Figure 2J), whose expression may thus be susceptible to external conditions. The existence of specific OMTs for these flavanones has been previously documented (Kim et al., 2006; Shimizu et al., 2012; Liu et al., 2013; Lee et al., 2017).

## Discussion

In this study, we tested whether herbarium plant specimens can provide a valuable resource for the discovery of new compounds with translational potential to treat AD. In collaboration with the SDNHM, we focused our investigation on the *Eriodictyon* genus, which includes *E. californicum*, a plant native to California that has been historically used as a medicine by local indigenous tribes.

The genus *Eriodictyon* comprises perennial herbs and shrubs restricted to the southwestern United States and northern Baja California, Mexico. Of the current 10 species belonging to the genus, four have been reported to be used for traditional medicine in the past (Bean and Saubel, 1972; Timbrook, 2007; Moerman, 2009; Wilken-Robertson, 2017). These include: *E. californicum*, predominantly grown in central/northern California and used by the Atsugewi, Costonoan, Kawaiisu, Mendocino Indian, Miwok, Pomo, Round Valley Indian, Yokut, Yuki, and Yurok tribes; *E. angustifolium*, grown in the southeastern part of California and used by the Paiute and Shoshone tribes; *E. trichocalyx*, common in southern California and used by the Cahuilla and Kumeyaay (Diegueno, Ipai, Tipai); and *E. crassifolium*, from the southwest used by the Chumash, Luiseno, and Kumeyaay (Diegueno, Ipai, Tipai). However, it is likely that other tribes with access to these species also used them for medicinal purposes. Our data show that all four of these species produce sterubin, and their extracts were among the most protective in our assays. It should be noted that the oxytosis and ferroptosis assays represent toxic insults that are characteristic of aging and, therefore, are not exclusive to the central nervous system. In addition, we show that the *Eriodictyon* extracts were anti-inflammatory in a manner that correlated with the amount of sterubin. Therefore, we can justifiably wonder whether the medicinal properties attributed to these plants by the indigenous tribes could be due to sterubin. This would certainly explain why different *Eriodictyon* species with distinct geographic distributions were of medicinal relevance to tribes across the state. Interestingly, sterubin has been reported in several other species not belonging to the *Eriodictyon* genus that also have a history of traditional medicine. Two of these include *Artemisia monosperma* (Abu-Niaaj et al., 1993) and *Thymus mastichina* (Gordo et al., 2012).

Eriodictyol and homoeriodictyol do not appear to be relevant to the therapeutic properties of *Eriodictyon*. The levels of eriodictyol in the plant specimens are extremely low and homoeriodictyol is neither neuroprotective nor anti-inflammatory (Fischer et al., 2019). However, both these flavanones appear to be good indicators of species identity, since their presence was consistently observed across the species. In fact, our data perfectly match a proposed phylogenetic tree for the genus *Eriodictyon* based on trichome morphology (Hannan, 1988). Specifically, it proposed splitting the genus into two groups: one group (*E. altissimum*, *E. angustifolium*, *E. californicum*, *E. capitatum*, *E. crassifolium*, and *E. trichocalyx*) lacking capitate trichomes, while the other (*E. sessilifolium*, *E. tomentosum*, *E. traskiae*) had capitate trichomes on various organs. Consistent with this classification, we found eriodictyol and homoeriodictyol in all species except *E. sessilifolium*, *E. tomentosum*, and *E. traskiae*. We also did not detect these flavanones in *E. lobbii* and *E. parryi*, which were only added to the genus *Eriodictyon* (from the genera *Nama* and *Turricula*, respectively) after the publication of the study, and whose trichomes have not been compared yet. Further analysis of eriodictyol and homoeriodictyol in additional specimens may clarify whether these flavanones can indeed be used to ascertain phylogenetic relationships in the future.

Flavonoid methylation on hydroxyl groups is a common event in plants that is catalyzed by highly regiospecific OMTs through transfer of a methyl group from *S*-adenosyl-L-methionine (SAM) to flavonoid substrates. Considering our data with the flavanones and their chemical structures, we propose a biosynthetic pathway for the generation of sterubin and homoeriodictyol from eriodictyol based on two distinct OMTs in the genus *Eriodictyon* (Figure 2J). In fact, several reports have identified the existence of regiospecific OMTs for these flavanones. This is the case of ROMT-9, an OMT from rice that can methylate the 3′-OH group of eriodictyol to generate homoeriodictyol (Kim et al., 2006; Liu et al., 2013), and of OsNOMT, another OMT from rice that can methylate the 7-OH group of eriodictyol to generate sterubin (Shimizu et al., 2012; Lee et al., 2017). The existence of a distinct 7OMT could explain why sterubin is only detected in some specimens within the same *Eriodictyon* species. It is possible that the expression of this 7OMT is regulated by external factors such as weather and geographic conditions. There were not sufficient specimens in our study to draw any conclusions on this regard, but the literature indicates that leaves of yerba santa plants may be more effective if harvested in the fall (Hedges and Beresford, 1886).

Undoubtedly, the most important finding from our study is that it is possible to screen for neuroprotective and anti-inflammatory activities using very small amounts of plant material that has been preserved in herbarium collections for long periods of time. In fact, we were surprised with how stable sterubin, eriodictyol and homoeriodictyol were in specimens that dated back all the way to 1878. Indeed, our most active extract came from *E. californicum* leaves that were collected in 1933. We did not measure the levels of sterubin in fresh material. However, it seems unlikely that the drying process alters sterubin since there was no correlation between the age of the dried leaves and the levels of sterubin. Although our method proved useful to identify non-volatile phytochemicals in herbarium specimens, such as the flavonoids described in this study, it may not be suitable for assaying volatile compounds such as essential oils and terpenoids that are sensitive to the drying process and herbarium preservation. Nonetheless, we are convinced that our results unlock new opportunities for the discovery of novel treatments not only for neurodegenerative diseases but also other diseases of aging. Our results thus provide additional value to herbarium collections and could open up a new approach to bioprospecting. Many herbaria keep additional material from the same preserved specimens for molecular biology studies that can also be used for screening in disease assays without damaging the collection. The herbarium collection at the SDNHM hosts nearly 275,000 specimens, and many of these have been used in the past as traditional medicines. Future studies are already being planned with other plants of medicinal interest. This work will be carried out as much as possible in full partnership with local institutions, traditional healers and communities in order to respectfully conduct research in the area of Indigenous Knowledge, assuring the intellectual property rights and the sharing of benefits that may arise as a result of the study of Californian medicinal plants.

In summary, we report the occurrence of sterubin in other species of the *Eriodictyon* genus and underscore its relevance as a major active compound, substantiating its therapeutic potential. Our study not only offers insight into the traditional use of yerba santa across indigenous tribes in California, but also demonstrates for the first time the use of herbarium collections for the discovery of novel therapeutic compounds for the treatment of neurodegenerative diseases.

## Data Availability Statement

All datasets generated for this study are included in the article/supplementary material.

## Author Contributions

PM and AP performed the experiments and edited the manuscript. WF and DS-C performed the experiments. ZL edited the manuscript. JR performed the experimental design and the experiments, and edited the manuscript. AC performed the experimental design and the experiments, analyzed the data, and wrote the manuscript.

## Conflict of Interest

The authors declare that the research was conducted in the absence of any commercial or financial relationships that could be construed as a potential conflict of interest.
